# Editorial

**Published:** 1880-02

**Authors:** 


### EDITORIAL.

The following, taken from the *Xenia Gazette* of January 9th, 1880, though not exactly in the line of *dental* surgery, may, if acted on, lesson the demand for *accidental* surgery, while its insertion here will show the readers of the *Register* that, though unable for physical labor, our old friend, Dr. Watt, still thinks, and this thought is of special value to those who handle horses. The article is as follows:

### IMPROVED HORSE-SHOE.

EDITOR GAZETTE: The musings of invalids often turn to poetry. Mine have taken to horse-shoes. How often the noble horse is disabled for life by slipping on ice! How often the ignoble rider or driver meets with a serious or even fatal accident, as a punishment for his failure to protect the faithful horse with sharp shoes. It is well known that the common shoe is worn smooth in a few days by use on gravel or Mac Adamized roads, and a storm of sleet then makes traveling dangerous to horse and man. In the solitude of the sick-room, I have devised a shoe that is a self-sharpener, that is, the horse sharpens it as he uses it, and thus it remains sharp till worn out. The structure is simple, and is as follows: Let the smith split the ordinary caulks at the heels of the shoe, and insert in each a small blade of steel, which is to be welded into the iron. Let the caulk be then finished by hammering into a wedge shape. A toe-piece is to be formed after the same manner, only it will, perhaps, be more convenient to weld the iron and steel pieces together, before attaching to the toe of the shoe. Both toe and caulks may be a little shorter than if made solely of iron; and the steel may be brought to an axe temper.

Now, the reader will at once see that, as iron wears away faster than steel, the wedge shaped iron in the caulk will wear away as the shoe is used, leaving the sharp projection of steel constantly exposed at the extremities of the caulks and toe, till the shoe is entirely worn out. The improved shoes can be seen in use on my horse, but their appearance is like that of the ordinary shoe.

The hint for this device was taken from an eastern patent, but the end sought is obtained by a process more simple, and totally different from that of the patent. I believe this shoe will be a blessing to both man and beast, and if so, a consciousness of the fact may cheer the lonely hours incident to invalid life.

GEO. WATT, M. D.

### MEMORIUM.

MR. EDITOR :—The following resolutions, expressive of the feelings of the members, passed at a meeting of the Chicago Dental Society, Jan. 5th, 1880, I was instructed to forward to your journal for publication.

Yours, truly,

M. S. DEAN, Cor. Sec.

WHEREAS: The painful intelligence has just reached us, that Dr. Samuel S. White has, with appalling suddenness, taken his leave of earth and earthly things; be it, therefore,

*Resolved*: That in his death the nation has lost one of its most energetic, useful and upright citizens, and that the dental profession has been bereft of its noblest benefactor and best friend; one who, in the prosecution of his extended enterprises, advanced the art, and by his liberality, fostered the Science of Dentistry.

*Resolved*: That this society is especially indebted for the many kind favors which he has bestowed upon it from its first organization up to the present time; and that its members deplore his loss, both as a personal friend, and as a gentleman who has been ever true and generous to the profession of his early adoption, and that we will cherish his many noble qualities in ever-grateful remembrance.

*Resolved*: That we extend to his bereaved family, our most sincere and heartfelt sympathy for the great affliction which they have sustained.

### IN MEMORY OF DR. S. S. WHITE.

The Faculty of the Baltimore College of Dental Surgery invited the Dental Practitioners of Baltimore City to meet in the College Building on Tuesday evening, January 6th, 1880, for the purpose of taking action relative to the death of Dr. White.

The meeting, which was largely attended, was called to order by Prof. F. J. S. Gorgas, when on motion, Dr. Henry H. Keech, was elected President, and Dr. J. Emory Scott, Secretary.

Prof. Gorgas read a sketch of the life of the late Dr. S. S. White, and also a letter on the same subject from Dr. Wm. H. Atkinson, of New York. Eulogistic remarks were made by the President, Dr. Keech, Dr. A. W. Sweaney and others, and on motion, a committee was appointed to draft solutions of respect to the memory of the deceased. The committee, which consisted of Drs. F. J. S. Gorgas, T. S. Waters, and A. P. Gore, reported the following, which were unanimously adopted:

WHEREAS: The Dental Practitioners of Baltimore City having been called together to pay a tribute of respect to the memory of the late Dr. Samuel Stockton White, of Philadelphia, whose death may be regarded as both a professional and public loss; therefore, be it

*Resolved*: That the Dental Practitioners of Baltimore City have learned, with deep regret, of the death of one who has been for many years, closely identified with the Dental Profession, at that period of life when the mental powers are in their fullest vigor, when his honorable career as an enterprising business man had won for him a world-wide reputation, and whose personal qualities, as exemplified in every relation of life, had secured the warm attachment and high respect of a large circle of devoted friends.

*Resolved*: That the Dental Practitioners of Baltimore City fully recognize the obligations American Dentistry owes to the late Dr. S. S. White, through the active agencies he has awakened for the elevation of the science, his devotion to its interest and the zeal, energy and intelligence he displayed in useful inventions and dental literature.

*Resolved*; That this expression of appreciation of the worth of the late Dr. Samuel Stockton White, in the form of a copy of these resolutions, be transmitted to the bereaved family of the deceased, and that they also be published in the different Dental Journals.

Respectfully submitted,

F. J. S. Gorgas,
T. S. Waters,
A. P. Gore,
*Committee.*

Professors Gorgas and Winder, on the part of the Faculty of the Baltimore College of Dental Surgery, and Dr. H. H. Keech, on the part of the Dental Practitioners of Baltimore City, were appointed to attend the funeral in Philadelphia.

### A NEW TOOTH BRUSH.

A new model of tooth brush devised by Dr. E. Pierrepont of Manchester, England, comes more nearly filling, all the requisites of such an implement, and anything heretofore presented.

The form is adapted, to the work to be accomplished, and is such as to be more acceptable in the mouth than any of the common firms. It is very desirable that it should be more extensively in the market.

### TRANSACTIONS OF THE CALIFORNIA STATE DENTAL ASSOCIATION.

A volume containing the proceedings of the last annual meeting of this body, has just been issued in pamphlet form, occupying three hundred pages. It is replete with interesting and instructive matter, none of which was published before its appearance in this volume. It has quite a number of valuable papers, and reports of much interesting discussions.

Some examination of the contents of this volume warrants the statements that the profession on the Pacific Coast, compairs favorably in association work with that on the Atlantic side of the country.

The subject of dental education is occupying the attention of the profession in California; and in a very practicable way—viz: in the establishment of a dental College, which in a short time will be in active operation.

An effort is also being made to secure a law regulating the practice of dentistry in that State.

With these, and such topics, the profession in California is expending labor and effort, and it will certainly not be without good results.

### ILLINOIS STATE DENTAL SOCIETY.

The Transaction of the last annual Session of this body is just at hand, full of interesting and instructive matter. This is one of the oldest and most active of our State Societies, embracing in it some of the best men in the profession.

At the meetings of this body there are always a number of good papers, and the discussions upon them are always interesting. Two of the papers read at the last meeting will be found in the pages of the present number of the Register.

### MISSISSIPPI VALLEY ASSOCIATION OF DENTAL SURGEONS.

The thirty-sixth annual metting of this Association will take place in the Dental College, Cincinnati, Ohio, on the first Tuesday in March, at ten o'clock A. M.

It is very desirable that the members should turn out in force and make the coming meeting the largest and most profitable in the history of the Society. E. G. Betty, Sec'y.

### NEW SCALERS.

Prof. A. O. Rawls of Lexington, Ky., has devised and just brought out a set of Scalers, consisting of nine instruments. These are more perfect in form and adaptation, than any that have been as yet presented to the profession, they are delicate and so formed as to operate upon any desired point of the teeth. There has to this time, been a greater deficiency in the adaptation of this class of instruments, than any other used by the dentist.

and great credit is due to him who makes such advance as is exhibited in this instance. I trust Prof. R. will remember that there is still room in some other directions.

### STUDY IT.

To the first article in the January number of the Register, viz: "Salivary Calculus and its Concomitants," I would invite special attention. If we will all study that paper and the principles involved—master them indeed—a long step will have been attained in the direction of progress.

This subject is of such importance, and so clearly set forth in this article, that no reader of the Register should pass it over with only a cursory reading. It possesses additional interest from the fact that Dr. Watt regards it as his farewell effort, in this direction at least, to the brethren of his loved profession. That this may not be his final good-by is the earnest wish and prayer of all who know him.

### OHIO DENTAL COLLEGE ASSOCIATION.

The annual meeting of this body will be held in the lecture room of the college, on Tuesday, March 2nd, 1880, at 2½ o'clock, p. m. It is hoped that all the members, so far as possible, will be present, and each do what he may for the interest and prosperity of the Institution.

There should be an endowed dental college in the West, and there is no better place for such an enterprise than Cincinnati, and a nucleus exists in the *Ohio Dental College*, to which, if there was an endowment attached sufficient to half support a good corps of teachers, the other half coming from the fees of students, and a liberal supply of all apparatus requisite for teaching, provided, its efficiency would be greatly increased.

The spirit of the time requires some definite advance movement on the part of some of our dental colleges, and it is to be hoped that this, one of the oldest, will be one of the first to take some such step.

### COMMENCEMENT.

The annual commencement of the *Ohio Dental College* will be held on the evening of Wednesday, March 3rd, in College Hall on Walnut street. The regular address of the evening will be given by Dr. M. S. Dean, of Chicago. This would seem to be enough to say as to the interest of the occasion; but there are other interesting and pleasant exercises. A general invitation is extended to all interested.

### THE MISSISSIPPI VALLEY DENTAL SOCIETY.

### THE THIRTY-SIXTH ANNUAL MEETING

OF THIS BODY WILL BE HELD IN THE LECTURE ROOM OF THE OHIO COLLEGE OF DENTAL SURGERY, ON TUESDAY, MARCH, 2ND, 1880, AT 10 O'CLOCK, A. M.

A cordial invitation is extended to all members of the profession to be present, and add to the interest, and profit of the occasion. It is important that every member be present.

The following subjects, will be presented for consideration at that time:

1st. Treatment and management of exposed tooth pulp.

2nd. Professional culture,—scientific and æsthetic.

3rd. Is it important that dental students should understand histological pathalogy?

4th. Is neuralgia a functional affection, or does it involve structural change of nerve tissue?

5th. Is the treatment of inflamed or sensitive dentine by escharotics in accordance with true physiology?

6th. Can thermal changes affect injuriously gold fillings?

7th. The progress of practical dentistry,—Operative,—Prosthetic.

8. With what therapeutic principles, is the true dentist more especially interested? Give examples of agents pertaining to each, with their mode of action.

*Executive Committee.* { J. TAFT,<br>J. TAYLOR,<br>A. O. RAWLS.

Page(s) missing

